# Assessing real-world safety concerns of Sacituzumab govitecan: a disproportionality analysis using spontaneous reports in the FDA adverse event reporting system

**DOI:** 10.3389/fonc.2023.1276976

**Published:** 2023-10-06

**Authors:** Xiujuan Gui, Jianli Zhao, Linxiaoxiao Ding, Jie Chai, Hongna Lai, Yangyang Cai, Simin Luo, Yinduo Zeng, Wenjing Wu, Haizhu Chen, Herui Yao, Ying Wang

**Affiliations:** Breast Tumor Center, Sun Yat-sen Memorial Hospital, Sun Yat-sen University, Guangzhou, Guangdong, China

**Keywords:** Sacituzumab govitecan, FAERS, safety, pharmacovigilance, pneumonitis, sepsis

## Abstract

**Aim:**

The aim of this study was to identify potential safety concerns associated with Sacituzumab Govitecan (SG), an antibody-drug conjugate targeting trophoblastic cell-surface antigen-2, by analyzing real-world safety data from the largest publicly available worldwide pharmacovigilance database.

**Methods:**

All data obtained from the FDA Adverse Event Reporting System (FAERS) database from the second quarter of 2020 to the fourth quarter of 2022 underwent disproportionality analysis and Bayesian analysis to detect and assess the adverse event signals of SG, considering statistical significance when the lower limit of the 95% CI >1, based on at least 3 reports.

**Results:**

Total of 1072 cases were included. The main safety signals were blood and lymphatic system disorders [ROR(95CI)=7.23 (6.43-8.14)], gastrointestinal disorders [ROR(95CI)=2.01 (1.81-2.22)], and relative infection adverse events, such as neutropenic sepsis [ROR(95CI)=46.02 (27.15-77.99)] and neutropenic colitis [ROR(95CI)=188.02 (120.09-294.37)]. We also noted unexpected serious safety signals, including large intestine perforation [ROR(95CI)=10.77 (3.47-33.45)] and hepatic failure [ROR(95CI)=3.87 (1.45-10.31)], as well as a high signal for pneumonitis [ROR(95CI)=9.93 (5.75-17.12)]. Additionally, age sub-group analysis revealed that geriatric patients (>65 years old) were at an increased risk of neutropenic colitis [ROR(95CI)=282.05 (116.36-683.66)], neutropenic sepsis [ROR(95CI)=101.11 (41.83-244.43)], acute kidney injury [ROR(95CI)=3.29 (1.36-7.94)], and atrial fibrillation [ROR(95CI)=6.91 (2.86-16.69)].

**Conclusion:**

This study provides crucial real-world safety data on SG, complementing existing clinical trial information. Practitioners should identify contributing factors, employ monitoring and intervention strategies, and focus on adverse events like neutropenic sepsis, large intestine perforation, and hepatic failure. Further prospective studies are needed to address these safety concerns for a comprehensive understanding and effective management of associated risks.

## 1 Introduction

The rapid development of antibody-drug conjugates (ADCs) has been accompanied by advancements in cancer biomarkers, individualized therapy, and pharmaceutical technology in recent years (1, 2). ADCs have demonstrated remarkable selectivity and efficacy, thereby revolutionizing cancer therapy pattern (2). Sacituzumab govitecan (SG) is a novel ADC, which exhibits promising results for the treatment of solid tumors, including breast cancer and urothelial cancer (3).

SG is a novel ADC that is a trophoblastic cell surface antigen-2 (TROP-2) directed antibody conjugated to the active metabolite of irinotecan, SN-38 (govitecan) (4). TROP-2 is highly expression in breast cancer, particularly in triple negative breast cancer. SG binds TROP-2 and delivers SN-38 to the tumor upon cleavage of the CL2A linker (5). SN-38 then interacts with topoisomerase I, preventing the re-ligation of topoisomerase I-induced single-strand breaks, ultimately leading to apoptosis and cell death (5). The ASCENT trial demonstrated the impressive efficacy of SG in patients with metastatic triple-negative breast cancer, with significantly longer progression-free survival and overall survival compared to single-agent chemotherapy (6).

Despite SG is a novel promising ADC, the potential safety risks have not yet been fully studied. To date, evidence on the safety profile of SG is primarily derived from its development programs and approval trials. The most frequently reported adverse events in these trials include neutropenia, nausea, diarrhea, fatigue, anemia, vomiting, rash, and constipation (6, 7). However, it is important to note that randomized controlled trials, despite their rigorous design, may not always capture unexpected or rare adverse events. This can be due to limitations such as short follow-up periods, variations in dosages, and differences between the enrolled study populations and the real-world population (8–10). Therefore, it becomes crucial to identify and assess potential safety concerns associated with SG in real-world settings. This will help clinicians gain a comprehensive understanding of the side effects of SG and subsequently improve the quality of life for patients.

Spontaneous reporting systems are critical in collecting and documenting adverse events related to pharmacotherapy in real-world settings and serve as a valuable resource for early identification of potential safety issues (11, 12). Pharmacovigilance and post-marketing analysis of spontaneous reporting databases have proven to be effective methods for detecting unexpected or rare adverse events. These databases serve as valuable sources of data that aid in the identification of safety concerns associated with pharmacotherapy, and they play a crucial role in improving drug safety in real-world settings. The FDA Adverse Event Reporting System (FAERS) is the largest publicly available pharmacovigilance database globally, making it an invaluable resource for accessing credible information on adverse events.

Therefore, the aim of this study was to analyze the real-world safety data of SG using the FAERS as an important complement of clinical trials.

## 2 Methods

### 2.1 Data source

The study collected and analyzed adverse events reports from the publicly available version of the FAERS database, covering the period from the second quarter of 2020 to the fourth quarter of 2022. Adverse events were categorized according to the Preferred Terms and classified into System Organ Classes (SOC) using the Medical Dictionary for Regulatory Activities version 25.0. The FAERS data files consist of seven databases, including demographic and administrative information, information on adverse drug reactions, drug information, drug therapy start and end dates, report source information, and indications for use/diagnosis.

Initially, the raw data underwent a deduplication process. Specifically, the latest FDA_DT (reported date) was selected among cases with the same CASEID (case ID), while the higher PRIMARYID (reported record ID) was chosen when both the CASEID and FDA_DT were identical. Next, we selected only adverse events where SG was assigned as either primary suspected. Lastly, to eliminate any potential interference from product-related issues, we excluded events related to product temperature excursion, device complaints, product container quality, product reconstitution quality, device dislocation, device occlusion, and device infusion issues from further analysis.

### 2.2 Study design and statistical analysis

**Figure 1:**
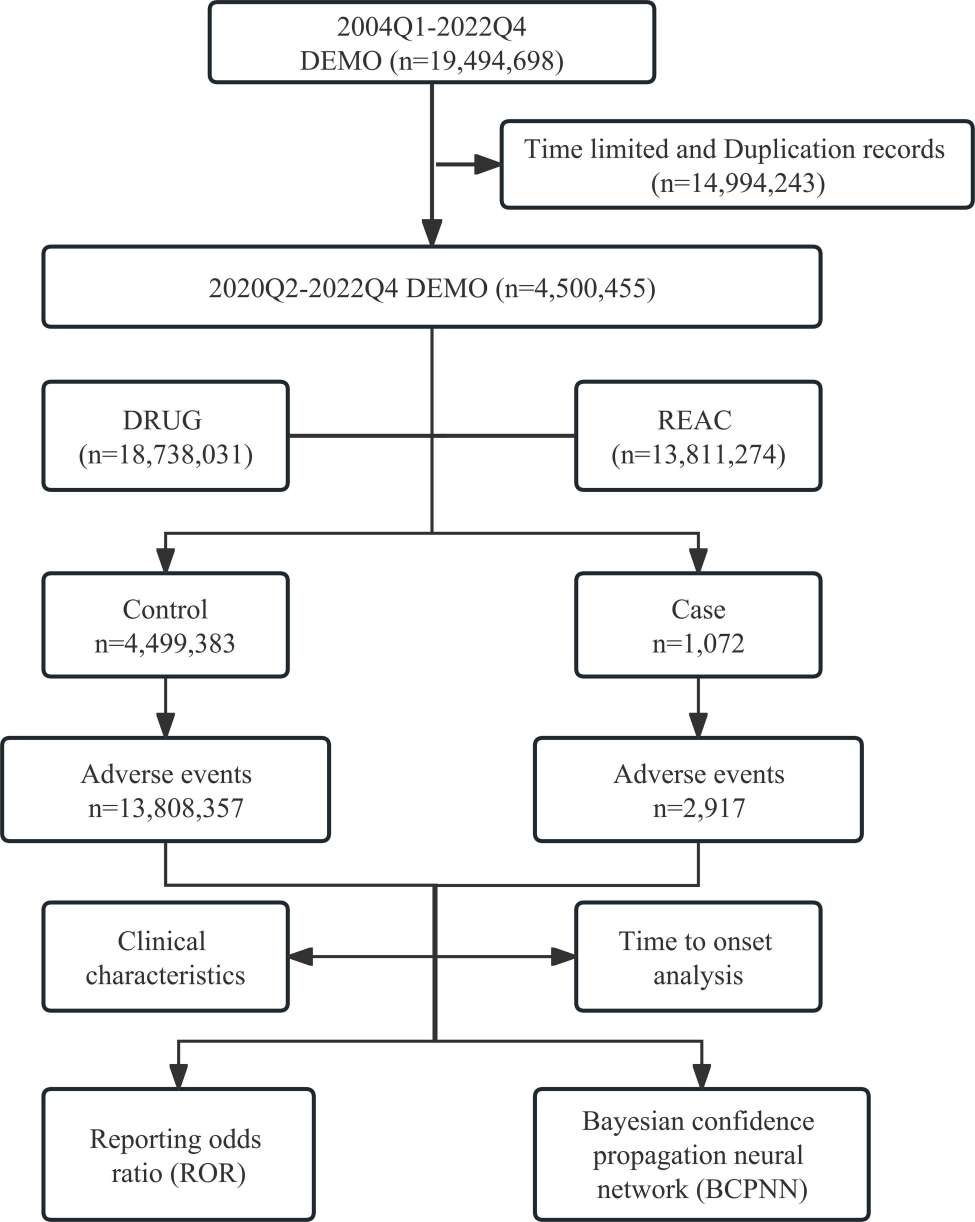
Flowchat of study.

As shown in **Figure 1**, we adopted the case/non-case approach, which is akin to the case-control study design (13, 14). Cases were defined as adverse event reports where “SACITUZUMAB GOVITECAN,” “TRODELVY,” or “IMMU-132” was recorded, whereas non-cases represented adverse event reports for all other drugs included in the FAERS database (10, 14). Disproportionality analysis represents a key tool of analytical methods in pharmacovigilance research, enabling the identification of drug-associated adverse events as signals by comparing the proportion of adverse events occurring between a specific drug and all other drugs (15). Within this cohort, we tested for disproportionality: if the proportion of adverse events of interest was found to be higher in patients exposed to SG (cases) than those not exposed (non-cases), an association could be hypothesized between the drug and the adverse event and considered a disproportionality signal (16). We performed reporting odds ratio (ROR) (17), and Bayesian confidence propagation neural network (BCPNN) analyses using information component (IC) as the signal index (18) to investigate the association between SG and adverse events. Traditional thresholds for significance were used (lower limit of the 95% CI >1 and >0 for ROR and IC, respectively), and a safety signal was considered if both approaches were statistically significant. Meanwhile, we compared the medicine specifications of SG in order to identify any unexpected adverse events. The selection of severe adverse events was based on the Designated Medical Event list provided by the European Medicines Agency.

**Table:** 

	Target AE	Other AEs
Target drug	a	b
Other drugs	c	d


$$\text{ROR}=\frac{\text{a}/\text{c}}{\text{b}/\text{d}}=\frac{\text{ad}}{\text{bc}}$$

$$ROR\;95CI=e^{\text{ln}\left(ROR\right)\pm 1.96\sqrt{\left(\frac{1}{a}+\frac{1}{b}+\frac{1}{c}+\frac{1}{d}\right)}}$$

$$\text{IC}=\log_{2}\frac{\text{a}\left(\text{a}+\text{b}+\text{c}+\text{d}\right)}{\left(\text{a}+\text{b}\right)\left(\text{a}+\text{c}\right)}$$

$$IC025=E\left(IC\right)-2\sqrt{Var\left(IC\right)}$$

The onset time was defined as the interval between EVENT_DT (date of adverse event occurrence) and START_DT (start date for SG use) (19). Moreover, input errors reports or inaccurate date entries were excluded. SAS v.9.4 and SPSS v.21 were used to data process and statistical analyses.

## 3 Results

### 3.1 Characteristics of cases treated with SG

A total 13,811,274 reports of adverse events were registered in FAERS, containing 2,917 SG-related adverse events in 1072 cases. **Table 1** provides a breakdown of the cases included in this study, with 905 female cases (84.4%), 78 male cases (7.3%), and 89 cases (8.3%) where gender information was not reported. The United States, France, and Canada were the top three countries reporting adverse events. Among the cases, 221 had a weight of less than 80 kg, 74 had a weight between 80-100 kg, 35 had a weight exceeding 100 kg, and 742 were of unknown weight. Additionally, 136 cases were older than 65 years, 398 cases were between 18-64 years old, and 538 cases did not report age information. 85.8% of the reporters were medical professionals. Lastly, the number of reported cases for the years 2020, 2021, and 2022 were 81, 384, and 607, respectively.

**Table 1:** Characteristics of all cases treated with sacituzumab govitecan.

Characteristic	N	%
Gender		
Female	905	84.4%
Male	78	7.3%
Not report	89	8.3%
Report Country		
United Stats	503	46.9%
France	177	16.5%
Canada	55	5.1%
Others	336	31.3%
Not report	1	0.1%
Weight (Kg)		
<80	221	20.6%
80-100	74	6.9%
>100	35	3.3%
Not report	742	69.2%
Age (year)		
>65	136	12.7%
18-64	398	37.1%
Not report	538	50.2%
Reporting Characteristic		
Health Profession	920	85.8%
Non-Health Profession	151	14.1%
Not report	1	0.1%
Reporting Year		
2020	81	7.6%
2021	384	35.8%
2022	607	56.6%

### 3.2 Disproportionality analysis of adverse events classified by SOC

**Figure 2:**
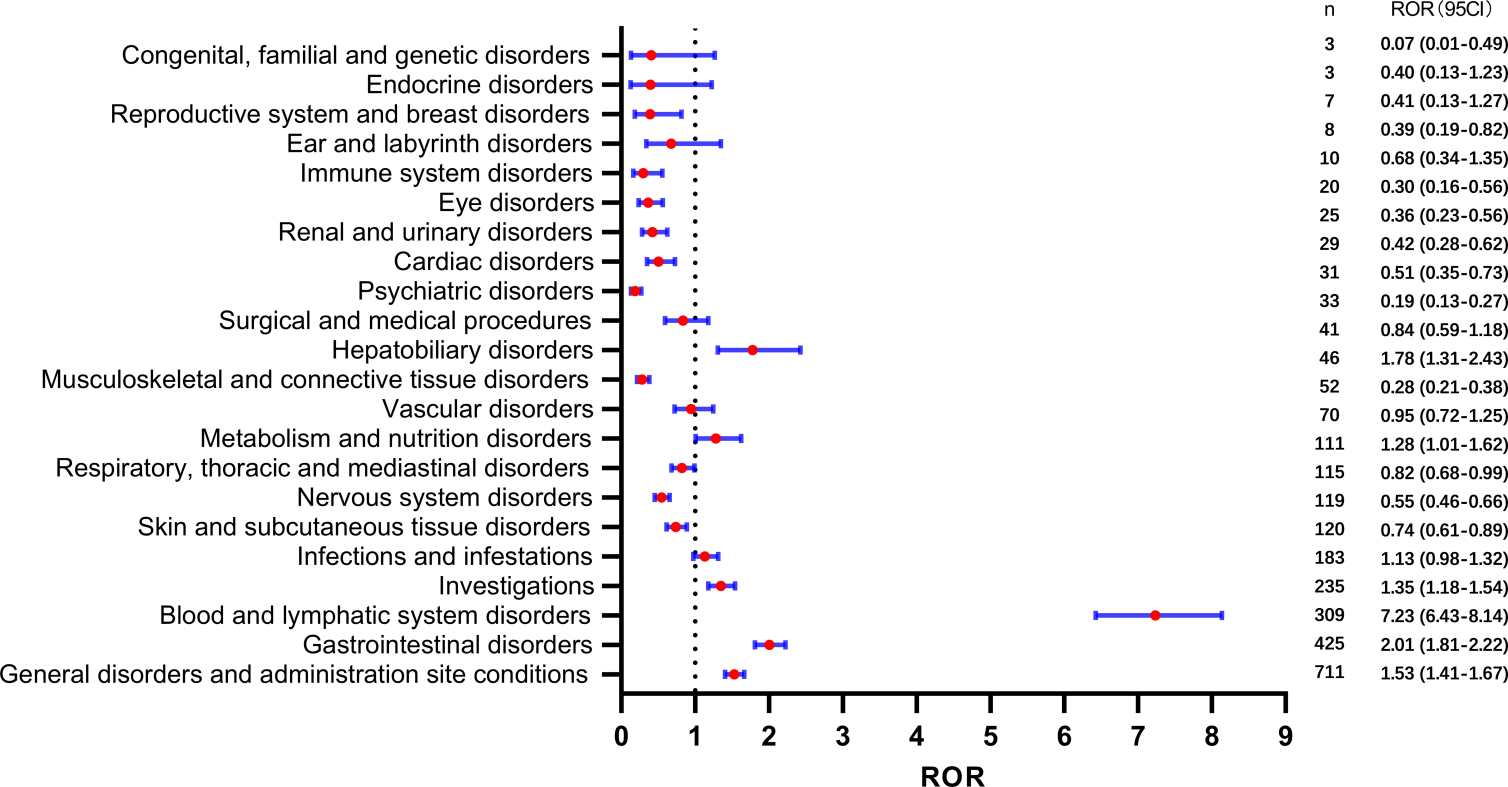
Safety signals of system organ class of adverse events.

We conducted a disproportionality analysis of the SOC to assess signal strengths and reports of SG. The results are presented in **Figure 2**. Our findings indicate that several SOCs exhibited strong signals, including hepatobiliary disorders [ROR(95CI)=1.78 (1.31-2.43)], metabolism and nutrition disorders [ROR(95CI)=1.28 (1.01-1.62)], investigations [ROR(95CI)=1.35 (1.18-1.54)], blood and lymphatic system disorders [ROR(95CI)=7.23 (6.43-8.14)], gastrointestinal disorders [ROR(95CI)=2.01 (1.81-2.22)], and general disorders and administration site conditions [ROR(95CI)=1.53 (1.41-1.67)]. Furthermore, the ROR (95CI) of infections and infestations was 1.13 (0.98-1.32); however, these findings did not reach statistical significance.

### 3.3 Disproportionality analysis of adverse events categorized according to the preferred terms

**Figure 3:**
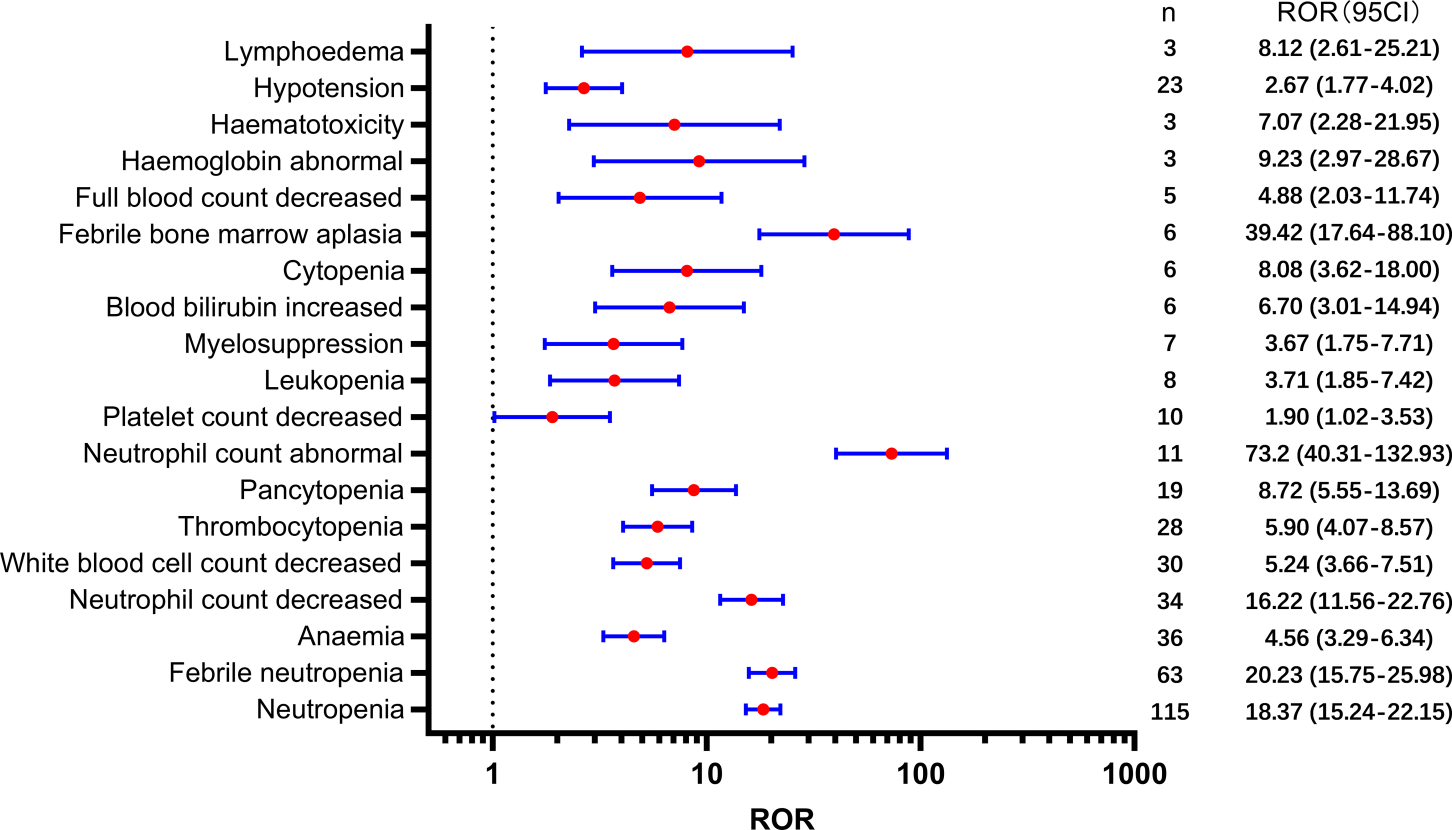
Significant safety signals of SG relative blood and lymphatic system disorders.

**Figure 4:**
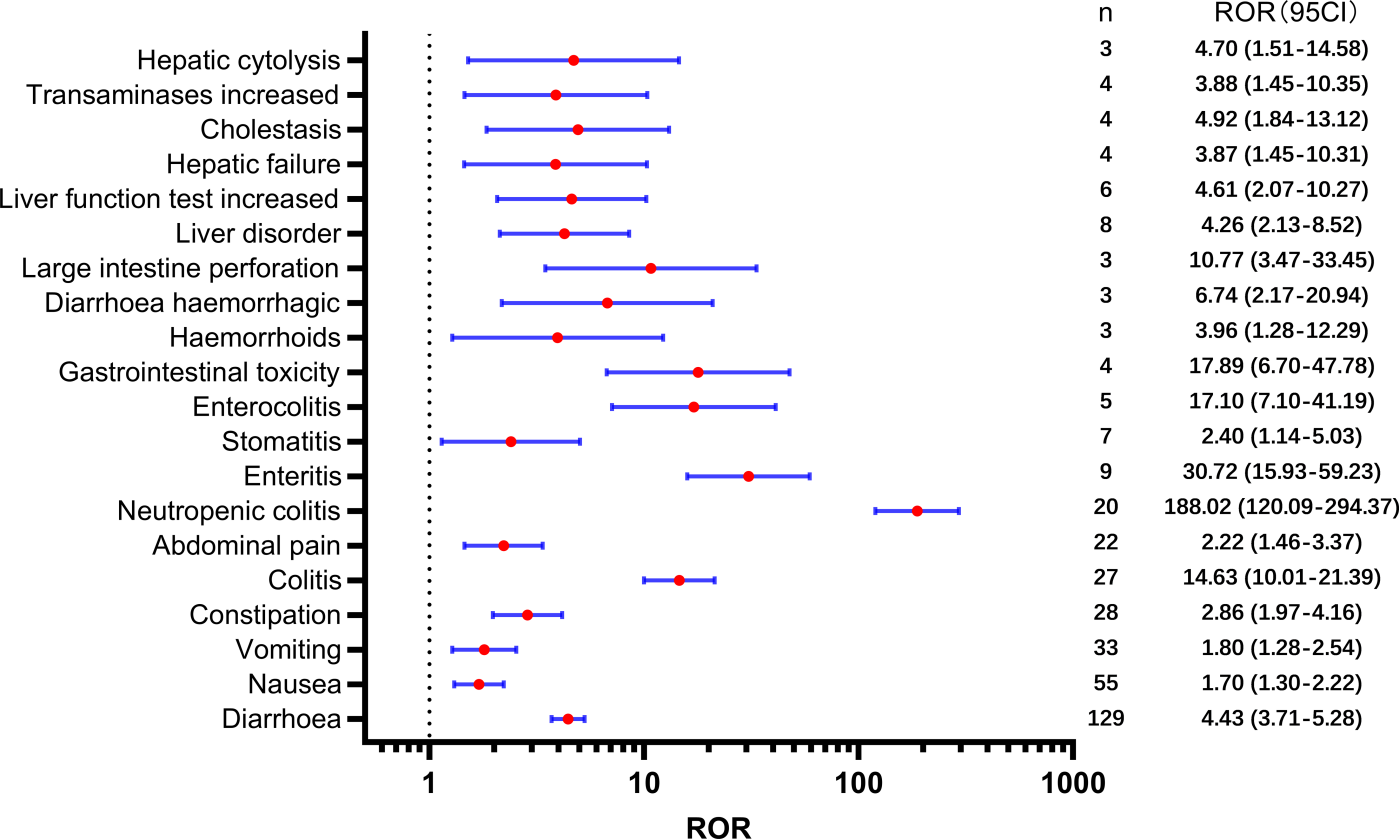
Significant safety signals of SG relative gastrointestinal and liver system disorders.

**Figure 5:**
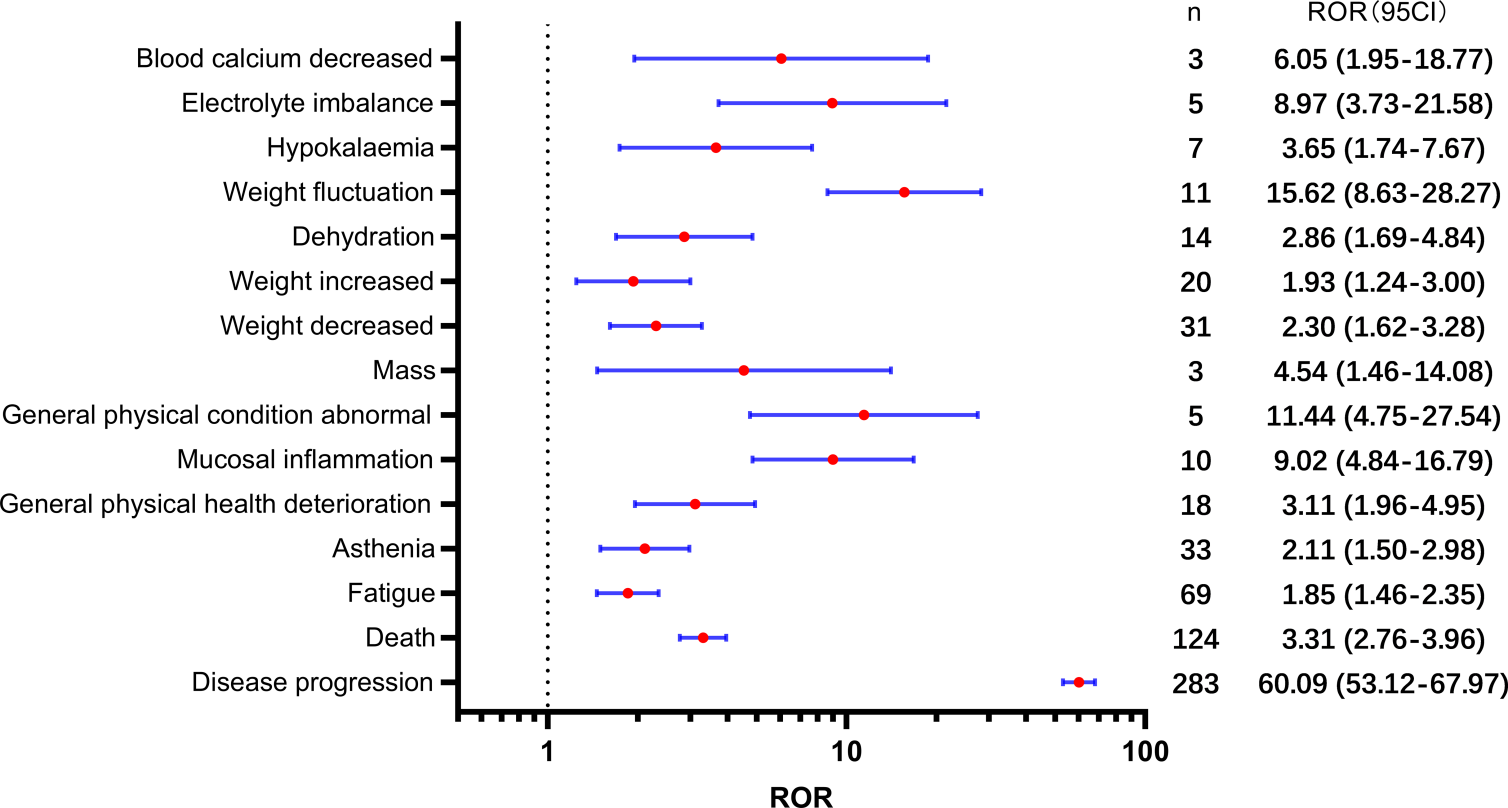
Significant safety signals of SG relative metabolism, nutrition, or general disorders.

**Figure 6:**
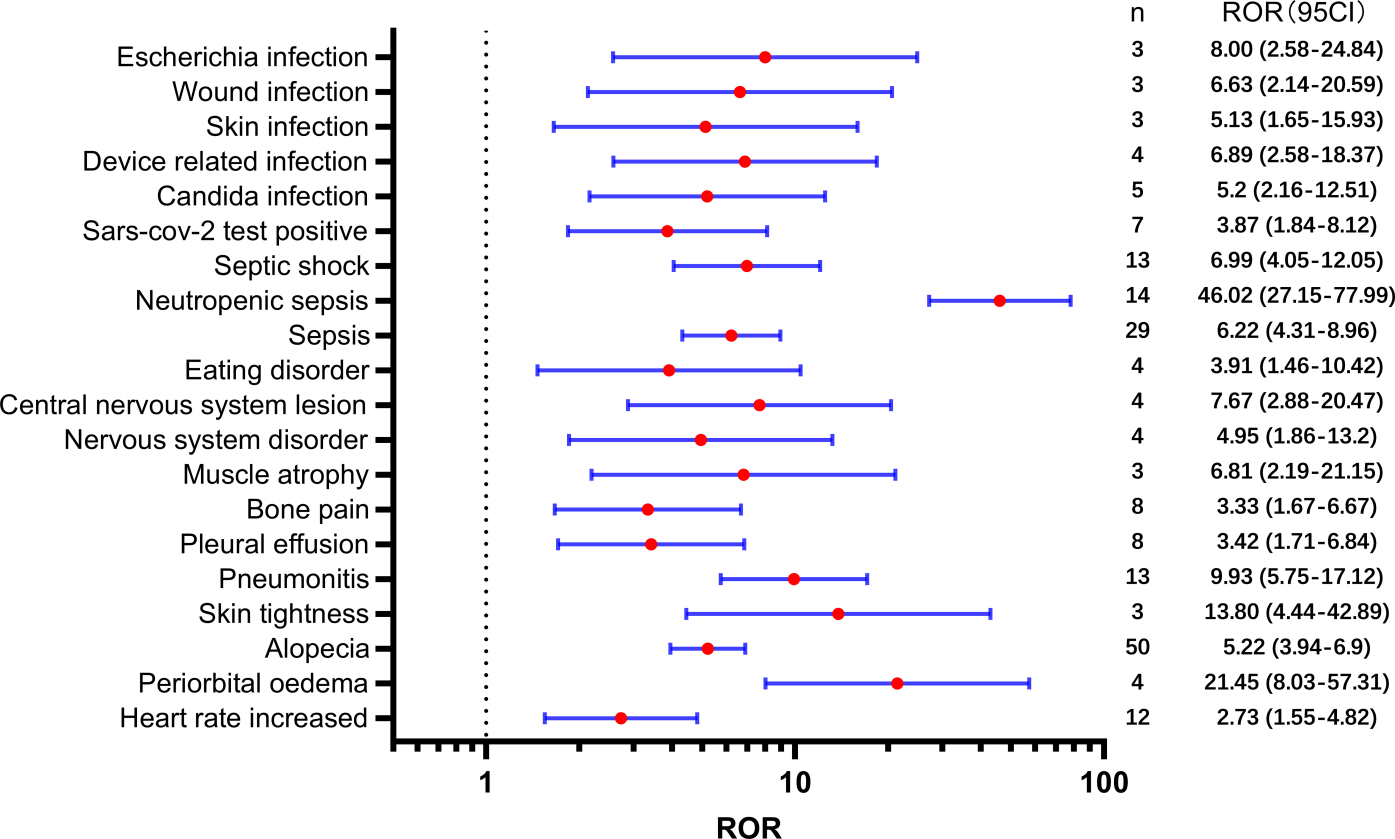
Significant safety signals of SG relative other system disorders.

**Table 2** presents a total of 75 adverse events with significant safety signals. Among these, febrile neutropenia [ROR(95CI)=20.23 (15.75-25.98)], neutropenic sepsis [ROR(95CI)=46.02 (27.15-77.99)], and neutropenic colitis [ROR(95CI)=188.02 (120.09-294.37)] were identified as three serious adverse events with high safety signals, excluding death and disease progression. Furthermore, we classified all significant adverse events in **Table 2** according to the SOC, which is presented in **Figures 3**–**5**, **6**. Specifically, for blood and lymphatic system-related adverse events (**Figure 3**), neutrophil count abnormal [ROR(95CI)= 73.20 (40.31-132.93)], febrile bone marrow aplasia [ROR(95CI)=39.42 (17.64-88.10)], and febrile neutropenia [ROR(95CI)=20.23 (15.75-25.98)] exhibited the highest ROR values. In terms of gastrointestinal and liver disorders (**Figure 4**), neutropenic colitis [ROR(95CI)=188.02 (120.09-294.37)], enteritis [ROR(95CI)=30.72 (15.93-59.23)], and gastrointestinal toxicity [ROR(95CI)=17.89 (6.70-47.78)] were identified as the top three ROR adverse events. Unexpected adverse events, including large intestine perforation [ROR(95CI)=10.77 (3.47-33.45)], hepatic failure [ROR(95CI)=3.87 (1.45-10.31)], cholestasis [ROR(95CI)=4.92 (1.84-13.12)], and hepatic cytolysis [ROR(95CI)=4.70 (1.51-14.58)], were also observed. For metabolism, nutrition, or general disorders (**Figure 5**), we found that disease progression [ROR(95CI)=60.09 (53.12-67.97)], weight fluctuation [ROR(95CI)=15.62 (8.63-28.27)], and general physical condition abnormal [ROR(95CI)=11.44 (4.75-27.54)] were the top three ROR adverse events. Lastly, **Figure 6** displays other adverse events, including skin, nervous system, or infection-related events such as pneumonitis [ROR(95CI)=9.93 (5.75-17.12)] and neutropenic sepsis[ROR(95CI)=46.02(27.15-77.99)], periorbital oedema [ROR(95CI)=21.45 (8.03-57.31)], and skin tightness [ROR(95CI)=13.80 (4.44-42.89)], which exhibited the highest ROR values. Although the pneumonitis showed high signal, interstitial lung disease didn’t show a significant signal [ROR(95CI)=1.35 (0.44-4.20), n=3].

**Table 2:** Safety signals of sacituzumab govitecan compared with all other drugs.

AE	n	ROR(95CI)	IC(IC025)
Disease progression	283	60.09 (53.12-67.97)	53.75(47.52)
Diarrhoea	129	4.43 (3.71-5.28)	4.27(3.58)
Death	124	3.31 (2.76-3.96)	3.21(2.68)
Neutropenia	115	18.37 (15.24-22.15)	17.63(14.62)
Fatigue	69	1.85 (1.46-2.35)	1.83(1.44)
Febrile neutropenia	63	20.23 (15.75-25.98)	19.74(15.37)
Nausea	55	1.70 (1.30-2.22)	1.69(1.29)
Alopecia	50	5.22 (3.94-6.90)	5.14(3.89)
Anemia	36	4.56 (3.29-6.34)	4.52(3.25)
Neutrophil count decreased	34	16.22 (11.56-22.76)	15.99(11.40)
Vomiting	33	1.80 (1.28-2.54)	1.79(1.27)
Asthenia	33	2.11 (1.50-2.98)	2.10(1.49)
Weight decreased	31	2.30 (1.62-3.28)	2.29(1.61)
White blood cell count decreased	30	5.24 (3.66-7.51)	5.20(3.62)
Sepsis	29	6.22 (4.31-8.96)	6.16(4.27)
Constipation	28	2.86 (1.97-4.16)	2.84(1.96)
Thrombocytopenia	28	5.90 (4.07-8.57)	5.85(4.03)
Colitis	27	14.63 (10.01-21.39)	14.46(9.90)
Hypotension	23	2.67 (1.77-4.02)	2.65(1.76)
Abdominal pain	22	2.22 (1.46-3.37)	2.21(1.45)
Weight increased	20	1.93 (1.24-3.00)	1.93(1.24)
Neutropenic colitis	20	188.02 (120.09-294.37)	179.69(114.77)
Pancytopenia	19	8.72 (5.55-13.69)	8.65(5.51)
General physical health deterioration	18	3.11 (1.96-4.95)	3.10(1.95)
Dehydration	14	2.86 (1.69-4.84)	2.85(1.69)
Neutropenic sepsis	14	46.02 (27.15-77.99)	45.37(26.77)
Septic shock	13	6.99 (4.05-12.05)	6.95(4.03)
Pneumonitis	13	9.93 (5.75-17.12)	9.87(5.72)
Heart rate increased	12	2.73 (1.55-4.82)	2.72(1.54)
Weight fluctuation	11	15.62 (8.63-28.27)	15.52(8.58)
Neutrophil count abnormal	11	73.20 (40.31-132.93)	71.84(39.56)
Platelet count decreased	10	1.90 (1.02-3.53)	1.89(1.02)
Mucosal inflammation	10	9.02 (4.84-16.79)	8.98(4.82)
Enteritis	9	30.72 (15.93-59.23)	30.44(15.79)
Bone pain	8	3.33 (1.67-6.67)	3.33(1.66)
Pleural effusion	8	3.42 (1.71-6.84)	3.41(1.70)
Leukopenia	8	3.71 (1.85-7.42)	3.70(1.85)
Liver disorder	8	4.26 (2.13-8.52)	4.24(2.12)
Stomatitis	7	2.40 (1.14-5.03)	2.39(1.14)
Hypokalemia	7	3.65 (1.74-7.67)	3.64(1.74)
Myelosuppression	7	3.67 (1.75-7.71)	3.66(1.74)
Sars-cov-2 test positive	7	3.87 (1.84-8.12)	3.86(1.84)
Liver function test increased	6	4.61 (2.07-10.27)	4.60(2.06)
Blood bilirubin increased	6	6.70 (3.01-14.94)	6.68(3.00)
Cytopenia	6	8.08 (3.62-18.00)	8.05(3.61)
Febrile bone marrow aplasia	6	39.42 (17.64-88.10)	39.02(17.46)
Full blood count decreased	5	4.88 (2.03-11.74)	4.87(2.02)
Candida infection	5	5.20 (2.16-12.51)	5.19(2.16)
Electrolyte imbalance	5	8.97 (3.73-21.58)	8.94(3.72)
General physical condition abnormal	5	11.44 (4.75-27.54)	11.40(4.74)
Therapy change	5	11.96 (4.97-28.79)	11.91(4.95)
Enterocolitis	5	17.10 (7.10-41.19)	17.02(7.07)
Hepatic failure	4	3.87 (1.45-10.31)	3.86(1.45)
Transaminases increased	4	3.88 (1.45-10.35)	3.87(1.45)
Eating disorder	4	3.91 (1.46-10.42)	3.90(1.46)
Cholestasis	4	4.92 (1.84-13.12)	4.91(1.84)
Nervous system disorder	4	4.95 (1.86-13.20)	4.94(1.85)
Device related infection	4	6.89 (2.58-18.37)	6.87(2.57)
Central nervous system lesion	4	7.67 (2.88-20.47)	7.65(2.87)
Gastrointestinal toxicity	4	17.89 (6.70-47.78)	17.80(6.66)
Periorbital oedema	4	21.45 (8.03-57.31)	21.33(7.98)
Hemorrhoids	3	3.96 (1.28-12.29)	3.95(1.27)
Mass	3	4.54 (1.46-14.08)	4.53(1.46)
Hepatic cytolysis	3	4.70 (1.51-14.58)	4.69(1.51)
Skin infection	3	5.13 (1.65-15.93)	5.12(1.65)
Blood calcium decreased	3	6.05 (1.95-18.77)	6.03(1.94)
Wound infection	3	6.63 (2.14-20.59)	6.62(2.13)
Diarrhoea hemorrhagic	3	6.74 (2.17-20.94)	6.73(2.17)
Muscle atrophy	3	6.81 (2.19-21.15)	6.80(2.19)
Hematotoxicity	3	7.07 (2.28-21.95)	7.05(2.27)
Escherichia infection	3	8.00 (2.58-24.84)	7.98(2.57)
Lymphoedema	3	8.12 (2.61-25.21)	8.10(2.61)
Hemoglobin abnormal	3	9.23 (2.97-28.67)	9.21(2.96)
Large intestine perforation	3	10.77 (3.47-33.45)	10.74(3.46)
Skin tightness	3	13.80 (4.44-42.89)	13.75(4.42)

### 3.4 Age sub-group analysis of adverse events

In the cohort of patients aged between 18 and 65 years, a total of 53 adverse events were identified with significant safety signals (**Table 3**), whereas in patients aged older than 65 years, 43 adverse events were reported (**Table 4**). Neutropenic colitis [ROR(95CI)=176.55 (91.15-341.97)], neutrophil count abnormal [ROR(95CI)=56.17 (20.99-150.28)], and neutropenic sepsis [ROR(95CI)=34.79 (14.44-83.85)] displayed the highest reporting odds ratios (RORs) among the top three adverse events in the 18-65 years old group. On the other hand, neutropenic colitis [ROR(95CI)=282.05 (116.36-683.66)], neutropenic sepsis [ROR(95CI)=101.11 (41.83-244.43)], and febrile bone marrow aplasia [ROR(95CI)=121.33 (38.9-378.43)] exhibited the highest ROR values among the top three adverse events in the >65 years old group. Notably, acute kidney injury [ROR(95CI)=3.29 (1.36-7.94)] and atrial fibrillation [ROR(95CI)=6.91 (2.86-16.69)] were identified as specific serious adverse events in patients aged over 65 years.

**Table 3:** Safety signals of sacituzumab govitecan in cases aged 18-65.

AE	n	ROR(95CI)	IC(IC025)
Disease progression	123	54.90 (45.6-66.10)	49.81(41.37)
Death	59	3.36 (2.59-4.36)	3.26(2.51)
Diarrhoea	58	4.23 (3.26-5.51)	4.10(3.15)
Neutropenia	42	14.15 (10.40-19.24)	13.73(10.09)
Nausea	37	2.46 (1.77-3.41)	2.42(1.75)
Fatigue	33	1.89 (1.34-2.67)	1.87(1.32)
Febrile neutropenia	32	21.91 (15.42-31.12)	21.38(15.05)
Alopecia	27	6.02 (4.11-8.82)	5.92(4.04)
Vomiting	20	2.33 (1.50-3.63)	2.31(1.49)
White blood cell count decreased	19	7.10 (4.52-11.18)	7.02(4.46)
Weight decreased	19	3.02 (1.92-4.75)	2.99(1.90)
Neutrophil count decreased	19	19.35 (12.30-30.44)	19.06(12.11)
Anaemia	18	4.87 (3.06-7.75)	4.82(3.02)
Constipation	14	3.05 (1.80-5.17)	3.03(1.79)
Weight increased	13	2.69 (1.56-4.64)	2.67(1.55)
Hypotension	12	2.97 (1.68-5.24)	2.95(1.67)
Abdominal pain	12	2.58 (1.46-4.56)	2.57(1.45)
Heart rate increased	10	4.87 (2.61-9.07)	4.84(2.60)
Thrombocytopenia	10	4.48 (2.41-8.35)	4.45(2.39)
Colitis	10	11.51 (6.18-21.45)	11.42(6.13)
Dehydration	9	3.93 (2.04-7.57)	3.91(2.03)
Platelet count decreased	9	3.65 (1.89-7.03)	3.63(1.89)
Neutropenic colitis	9	176.55 (91.15-341.97)	172.42(89.01)
Weight fluctuation	8	24.26 (12.10-48.65)	24.07(12.00)
Pancytopenia	7	6.83 (3.25-14.36)	6.80(3.23)
Liver function test increased	6	9.85 (4.41-21.97)	9.80(4.39)
Septic shock	6	6.87 (3.08-15.33)	6.84(3.07)
Bone pain	6	5.34 (2.39-11.91)	5.32(2.38)
Pneumonitis	5	8.12 (3.37-19.56)	8.09(3.36)
Pleural effusion	5	4.56 (1.89-10.97)	4.54(1.89)
Neutropenic sepsis	5	34.79 (14.44-83.85)	34.55(14.34)
Stomatitis	5	3.65 (1.52-8.79)	3.64(1.51)
Gastrooesophageal reflux disease	5	3.28 (1.36-7.88)	3.27(1.36)
Candida infection	5	11.11 (4.61-26.74)	11.06(4.59)
Blood bilirubin increased	4	9.54 (3.57-25.45)	9.50(3.56)
Neutrophil count abnormal	4	56.17 (20.99-150.28)	55.70(20.82)
SARS-CoV-2 test positive	4	4.71 (1.77-12.57)	4.70(1.76)
Throat irritation	4	4.61 (1.73-12.30)	4.60(1.72)
Hypokalemia	4	4.45 (1.67-11.88)	4.44(1.66)
Leukopenia	4	3.95 (1.48-10.55)	3.94(1.48)
Enteritis	4	29.01 (10.86-77.51)	28.85(10.80)
Oxygen saturation decreased	4	2.67 (1.00-7.13)	2.66(1.00)
Hemorrhoids	3	8.45 (2.72-26.26)	8.43(2.71)
Cholestasis	3	7.87 (2.53-24.44)	7.85(2.53)
Hypersomnia	3	5.16 (1.66-16.03)	5.15(1.66)
Neuralgia	3	5.12 (1.65-15.90)	5.11(1.65)
Pain of skin	3	3.79 (1.22-11.76)	3.78(1.22)
Limb discomfort	3	3.66 (1.18-11.38)	3.66(1.18)
Liver disorder	3	3.40 (1.09-10.56)	3.39(1.09)
Myelosuppression	3	3.35 (1.08-10.41)	3.35(1.08)
Enterocolitis	3	21.86 (7.03-67.96)	21.77(7.01)
Muscle atrophy	3	14.54 (4.68-45.18)	14.49(4.66)
Wound infection	3	14.16 (4.56-44.00)	14.11(4.54)

**Table 4:** Safety signals of sacituzumab govitecan in cases aged >65.

AE	n	ROR(95CI)	IC(IC025)
Diarrhoea	22	4.65 (3.03-7.14)	4.48(2.92)
Disease progression	18	21.84 (13.64-34.99)	21.04(13.13)
Febrile neutropenia	16	31.92 (19.39-52.56)	30.85(18.73)
Sepsis	15	20.22 (12.09-33.82)	19.60(11.72)
Neutropenia	14	13.58 (7.98-23.12)	13.20(7.76)
Fatigue	11	1.82 (1.00-3.30)	1.80(0.99)
Thrombocytopenia	9	11.78 (6.09-22.79)	11.57(5.98)
Asthenia	8	3.17 (1.57-6.37)	3.13(1.56)
Anaemia	8	6.27 (3.12-12.61)	6.18(3.07)
Death	7	1.12 (0.53-2.36)	1.12(0.53)
Hypotension	7	5.03 (2.38-10.61)	4.97(2.36)
Alopecia	6	3.83 (1.71-8.58)	3.80(1.70)
Colitis	6	20.03 (8.95-44.84)	19.78(8.84)
Vomiting	5	1.68 (0.69-4.05)	1.67(0.69)
Weight decreased	5	2.28 (0.95-5.52)	2.27(0.94)
Weight increased	5	2.98 (1.24-7.20)	2.96(1.23)
Acute kidney injury	5	3.29 (1.36-7.94)	3.27(1.35)
General physical health deterioration	5	5.34 (2.21-12.89)	5.29(2.19)
White blood cell count decreased	5	5.38 (2.23-12.98)	5.33(2.21)
Atrial fibrillation	5	6.91 (2.86-16.69)	6.85(2.84)
Septic shock	5	16.63 (6.89-40.15)	16.45(6.82)
Neutropenic sepsis	5	101.11 (41.83-244.43)	99.72(41.25)
Neutropenic colitis	5	282.05 (116.36-683.66)	276.45(114.05)
Pain	4	0.69 (0.26-1.84)	0.69(0.26)
Dyspnoea	4	0.99 (0.37-2.64)	0.99(0.37)
Rash	4	1.21 (0.45-3.23)	1.21(0.45)
Abdominal pain	4	2.48 (0.93-6.64)	2.47(0.92)
Nausea	3	0.56 (0.18-1.75)	0.57(0.18)
Condition aggravated	3	1.04 (0.33-3.23)	1.04(0.33)
Pyrexia	3	1.18 (0.38-3.67)	1.18(0.38)
Constipation	3	1.88 (0.60-5.85)	1.88(0.60)
Somnolence	3	2.23 (0.72-6.95)	2.22(0.71)
Urinary tract infection	3	2.28 (0.73-7.10)	2.27(0.73)
Hemoglobin decreased	3	4.34 (1.39-13.50)	4.32(1.39)
Myocardial infarction	3	4.43 (1.42-13.78)	4.41(1.42)
Stress	3	4.91 (1.58-15.27)	4.88(1.57)
Dysphagia	3	5.19 (1.67-16.15)	5.16(1.66)
Leukopenia	3	8.59 (2.76-26.72)	8.54(2.74)
Speech disorder	3	8.70 (2.79-27.07)	8.65(2.78)
Pneumonitis	3	14.10 (4.53-43.9)	14.01(4.50)
Mucosal inflammation	3	16.68 (5.36-51.92)	16.57(5.32)
Eating disorder	3	18.12 (5.82-56.41)	18.00(5.78)
Febrile bone marrow aplasia	3	121.33 (38.9-378.43)	120.07(38.50)

### 3.5 Time to onset analysis of SG

**Figure 7:**
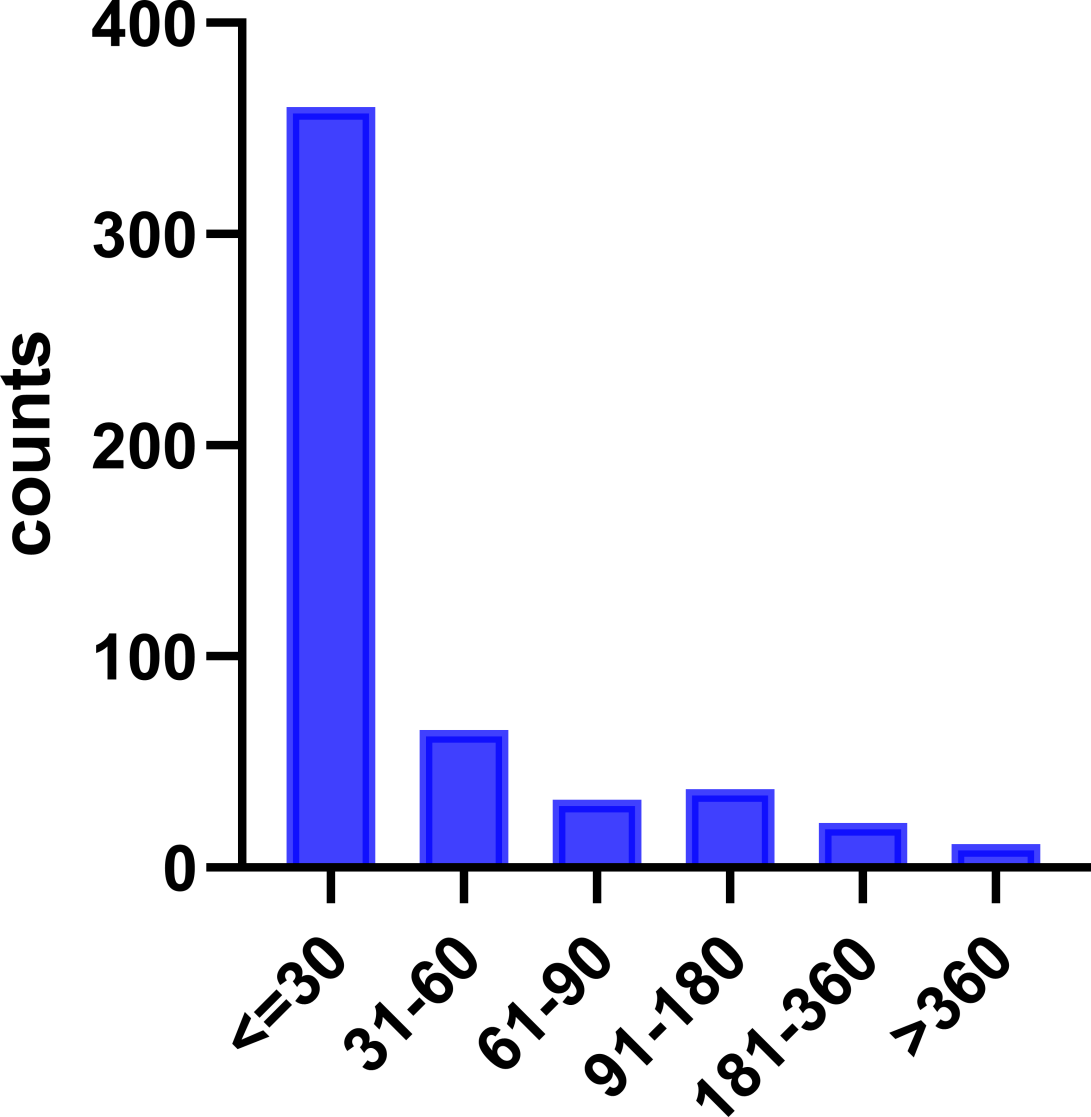
Time to onset of SG.

After excluding the reports with inaccurate, missing, or unknown onset time, a total of 526 adverse events were analyzed for their respective onset times. As shown in **Figure 7**, 360 reports (68.4%) indicated that the adverse events occurred within 30 days of drug administration. Over 80% of the adverse events were observed within 60 days of drug administration, while only a minor proportion of 2.1% reported onset times longer than 360 days.

## 4 Discussion

To our knowledge, this pharmacovigilance study, based on the FAERS database, presents the first real-world safety profile of SG. The study highlights various safety concerns related to SG use and suggests a potential for serious and unexpected adverse events. Four main findings emerged from this study.

First, blood and lymphatic system disorders, along with gastrointestinal disorders, were the most frequently reported adverse events associated with SG use and showed significant high safety signals (**Figure 2**). Neutropenia, febrile neutropenia, and anemia were the most frequently reported adverse events in this SOC category. Consistent with the clinical trial findings, SG showed a high number of reports of neutropenia and febrile neutropenia (6, 20–22). Neutropenia is a common reason for treatment interruption (6), and it is a known toxicity associated with irinotecan and SN-38 (23). Notably, our analysis revealed a high safety signal for neutropenia-related adverse events, including neutropenic colitis and neutropenic sepsis. Several infection-related adverse events, such as sepsis, septic shock, and skin infection, also showed a high safety signal. Considering that neutropenia or myelosuppression increases the risk of infection, it is important to consider the use of granulocyte colony-stimulating factor, which helps stimulate the production of white blood cells in the bone marrow (24, 25). Additionally, it is crucial to address contributing factors that can mitigate the risk of infection-related complications, particularly neutropenic sepsis and sepsis. In such cases, appropriate anti-infective therapy should be considered. Furthermore, it is worth noting that neutropenic colitis, although rare, has shown significant signals. To manage neutropenic colitis effectively, strategies such as bowel rest, intravenous rehydration therapy, and the administration of broad-spectrum antibiotics may be beneficial. These interventions can help alleviate symptoms and promote recovery from neutropenic colitis (26).

Regarding gastrointestinal disorders, diarrhea was the most frequently reported adverse event associated with SG, which is consistent with regulatory trials (6). SG-related diarrhea was also associated with other adverse events, such as electrolyte imbalance, hypokalemia, hypocalcemia, and dehydration, all of which showed a high safety signal. Approximately 9% of patients experienced severe diarrhea, for which loperamide and supportive measures are recommended (6, 27). Additionally, nausea, vomiting, and constipation, as well as enteritis, colitis, and enterocolitis, were associated with high safety signals. Importantly, we identified an increased risk of large intestine perforation. It is hypothesized that SG-induced diarrhea may lead to weakened mucosal integrity and injury (28), which could potentially result in this serious adverse event. However, it is important to approach this unexpected finding with caution, as it is based on a limited number of reported cases (only three cases were reported). Given the small sample size, further investigation and analysis are necessary to gain a clearer understanding of the implications and significance of this finding.

Second, the potential hepatobiliary toxicity associated with SG warrants increased attention. Our analysis identified unexpected adverse events such as hepatic failure, cholestasis, and hepatic cytolysis. The liver and kidneys are most at risk due to systemic exposure to the unconjugated cytotoxic payload (29). Studies have reported that irinotecan-induced hepatotoxicity is mainly characterized by an increase in transaminase levels and the development of steatosis. Several significant risks and predisposing factors for this condition have been identified, including high body mass index or obesity, diabetes, and a high-fat diet (30). Furthermore, the potential mechanisms underlying hepatic injury induced by the cytotoxic payload of irinotecan include the expression of TROP-2 by normal cells, internalization of the payload by liver sinusoidal endothelial cells and Kupffer cells through endocytosis, and impairment of disposition pathways. To better understand the underlying mechanisms of drug-induced liver injury, further research is needed at the cellular and molecular levels.

Third, our analysis identified a high incidence of pneumonitis associated with SG. It is noteworthy, however, that only two cases of pneumonitis were reported in the Phase I-III clinical trials of SG (6, 7, 22, 31). The signal could potentially be due to a variety of factors, such as COVID-19 infection risk, increased risk of infection due to SG treatment, or interstitial lung disease induced by ADC. ADCs have been shown to induce interstitial lung disease, particularly trastuzumab deruxtecan, which resulted in 10.5% of patients experiencing interstitial lung disease or pneumonitis (32). A meta-analysis evaluated the incidence of ADC-associated pneumonitis and found that the total incidence of all-grade drug-associated pneumonitis was higher in ADCs (33). In our study, we did not observe significant safety concerns related to interstitial lung disease, which may indicate a potential advantage of SG compared to other ADCs. However, it is important to note that the number of reported cases of interstitial lung disease was limited in our study (n=3), which hinders a robust assessment of the risk. Although we did not observe significant safety concerns related to interstitial lung disease in our study, it is essential to consider the inherent limitations of our sample size. Therefore, further long-term monitoring of interstitial lung disease should be conducted to better understand the potential advantage of SG compared to other ADCs in terms of this particular adverse event

Fourth, it is widely recognized that older age is a risk factor for chemotherapy toxicity (23), which not only affects treatment decisions but also increases the risk of adverse events. In this study, there were 50% of the reports lack of age data, this may lead to the bias of the results. Compared with other studies, our findings were consistent with those of a study conducted by Rugo et al., which suggested that the safety profile of SG in patients aged ≥65 was generally similar to that of patients aged <65 and the overall study population (27). However, our analysis also revealed that geriatric patients (>65 years old) exhibited high signals of sepsis, febrile bone marrow aplasia, and unexpected adverse events, including acute kidney injury and atrial fibrillation. These signals indicated that the potential risk of infection, kidney injury, and cardiotoxicity was increased in geriatric patients. There are several factors likely to increase the risk of ADC-related adverse events, such as age-related changes in homeostasis and physiological conditions (34–36), as well as age-related pharmacokinetic changes (37, 38). It is crucial to monitor the safety of SG in geriatric patients, particularly those with renal lesions or cardiac diseases.

There are several limitations to this study that should be acknowledged. First, it is not possible to confirm a causal relationship between adverse events and SG administration based on data from FAERS. Future clinical studies may utilize the Naranjo Adverse Drug Reaction Probability Scale to assess whether there is a causal relationship between an adverse event and the drug (39). Second, it is important to note that the unavoidable bias in this study cannot be eliminated. In general, safety signals may be biased by unmeasured confounding factors, such as height, weight, age, drug co-administration, or the knowledge and attitudes of healthcare professionals (10). For example, certain demographic or clinical characteristics (such as age, gender, or pre-existing medical conditions) might influence the reporting patterns and frequencies of adverse events, potentially leading to biased results when using the signal algorithm. Third, the absence of age data in the database can introduce bias when conducting age subgroup analysis. Fourth, the safety signals identified in this study may have been affected by cancer symptoms or other treatments. Inclusion of such symptoms in the analysis can affect the interpretation and analysis of the data, potentially impacting the overall findings and conclusions of the study. Fifth, as SG is a newly approved drug (April 2020), the quantity of reports in special populations was not sufficient. Longer-term and continuous monitoring is necessary to explore the potential risks and benefits of SG.

## 5 Conclusion

This analysis, based on real-world pharmacovigilance data from the FAERS database, revealed significant safety signals associated with SG. The main safety concerns of SG align with those observed in clinical trials and involve blood and lymphatic system disorders, gastrointestinal disorders, and associated infections. Notably, rare, unexpected, and serious safety signals such as large intestine perforation and hepatic failure were also identified. To mitigate these adverse events, practitioners should identify potential contributing factors and implement appropriate monitoring and intervention strategies. This study provides important real-world safety data for SG, complementing existing findings from clinical trials. The identified safety concerns warrant further prospective investigation to ensure comprehensive understanding and management of these risks.

## Data availability statement

The original contributions presented in the study are included in the article/supplementary material. Further inquiries can be directed to the corresponding authors.

## Author contributions

XG: Data curation, Formal Analysis, Investigation, Writing – original draft, Writing – review & editing. JZ: Data curation, Investigation, Writing – review & editing. LD: Data curation, Investigation, Writing – review & editing. JC: Data curation, Writing – review & editing. HL: Data curation, Writing – review & editing. YC: Investigation, Writing – review & editing. SL: Investigation, Writing – review & editing. YZ: Investigation, Writing – review & editing. WW: Investigation, Writing – review & editing. HC: Investigation, Supervision, Writing – review & editing. HY: Formal Analysis, Project administration, Resources, Supervision, Writing – review & editing. YW: Data curation, Formal Analysis, Project administration, Resources, Supervision, Validation, Writing – review & editing.
